# Anterolateral versus modified posterolateral approach for tibial plateau fractures with involvement of the posterior column: a cadaveric study

**DOI:** 10.1007/s00068-022-02113-8

**Published:** 2022-09-28

**Authors:** Peter Behrendt, Markus T. Berninger, Grégoire Thürig, Julius Dehoust, Jan H. Christensen, Karl-Heinz Frosch, Matthias Krause, Maximilian J. Hartel

**Affiliations:** 1 Department of Trauma Surgery, Orthopedics and Sports Orthopedics, Asklepios St. Georg, Hamburg, Germany; 2 Department of Trauma and Orthopaedic Surgery, University Medical Center Hamburg-Eppendorf, Hamburg, Germany; 3 Department of Anatomy, Christian-Albrechts-University, Kiel, Germany; 4 Department of Orthopedics and Traumatology, Cantonal Hospital Fribourg, Fribourg, Switzerland; 5 Department of Trauma Surgery, Orthopaedics and Sports Traumatology, BG Hospital Hamburg, Hamburg, Germany; 6 Department of Orthopedics and Traumatology, University Medical Center Schleswig-Holstein, Campus Kiel, Kiel, Germany

**Keywords:** Tibia, Fracture, Modified, Prone, Supine, Posterolateral approach

## Abstract

**Introduction:**

The aim of this study was to compare the reduction quality of the anterolateral (AL) and modified posterolateral approach (PL) in lateral tibial plateau fractures involving the posterior column and central segments.

**Methods:**

Matched pairs of pre-fractured cadaveric tibial plateau fractures were treated by either AL approach (supine position) or PL approach (prone position). Reduction was controlled by fluoroscopy and evaluated as satisfying or unacceptable. Afterwards, the reduction was examined by 3D scan.

**Results:**

10 specimens (3 pairs 41B3.1, 2 pairs 41C3.3) were evaluated. PL approach achieved significantly (*p* 0.00472) better fracture reduction results (0.4 ± 0.7 mm) of the posterior column compared to the AL group (2.1 ± 1.4 mm). Fracture steps involving the central area of the lateral plateau were insufficiently reduced after fluoroscopy using both approaches.

**Conclusion:**

Optimal reduction of displaced tibial plateau fractures involving the posterolateral column necessitates a posterior approach, which can be conducted in prone or lateral positioning. The anterolateral approach is indicated in fractures with minor displacement of the posterolateral rim but fracture extension in the latero-central segments. In these cases, an additional video-assisted reduction or extended approaches are helpful.

## Introduction

Tibial plateau fractures annually account for 10.3 per 100,000 fractures in an unselected patient population [1]. The most common type of fractures is AO type 41-B3 and 41-C3, which typically involve the postero-lateralo-central segment [2, 3]. Recent literature has focused on the biomechanical and clinical importance of proper reduction of this segment. Multiple studies have demonstrated that poor reduction in tibial plateau fractures result in post-traumatic deformities and osteoarthritis, significantly impairing functional outcomes and quality of life. A postoperative intraarticular joint irregularity of less than 2.5 mm results in a significantly improved range of motion, less pain and higher KOOS Score [3–5]. In addition, the posterolateral compartment seems to have implications for the joint kinematics and ligamentous stability [6, 7].

With respect to its clinical treatment by open reduction and internal fixation, an analysis of postoperative computed tomographies revealed up to 32.3% malreductions, that were mainly localized in the posterior quadrants of the lateral plateau [8]. The same authors found that neither age, nor body mass index, type of fracture or usage of locking plates were predictive of malreduction. In the literature, limited intraoperative visualization is accused of being the main reason for insufficient intraarticular reduction and sole fluoroscopic-controlled reduction is associated with malreduction [8, 9]. In cases of aforementioned type B and C fractures with involvement of the lateral plateau, the classic anterolateral approach only provides access to 36.6% of the anterior and lateral articular surface [10], which still yielded 16.6% malreductions [8]. Improved visualization can be achieved by an additional osteotomy of the lateral femoral epicondyle (more than 80% of the lateral plateau can be visualized) and a modified posterolateral approach providing additional access to the posterior tibial plateau [10–13]. With the patient positioned in prone position, the posterolateral approach enables a simultaneous posteromedial approach in cases of concomitant medial plateau fractures allowing at least for 270° accessibility of the tibial plateau.

The main scope of this study was a validation of improved reduction quality in tibial plateau fractures by two different approaches to the lateral tibia plateau. In a cadaveric study, the anterolateral approach in supine position of the specimens was compared with the modified posterolateral approach in prone position. It was hypothesized that improved visualization via the modified posterolateral approach yields improved fracture reduction of lateral tibial plateau fractures with involvement of the posterior lateral column.

## Materials and methods

### Study patients

Ten human cadaveric knee joints with artificially pre-fractured tibia plateau fractures involving the postero(lateral) column were examined. Pre-fractured knee joints with intact soft tissues were provided by Rimasys GmbH (Cologne, Germany). Inclusion criteria for this study were an involvement of one or both posterolateral segments (PLL = postero-latero-lateral, PLC = postero-latero-central) according to the 10-segment classification. Fractures were classified by two independent senior orthopedic residents according to OTA/AO and 10-segment classification. Mean age of the specimens was 65 ± 8.4 years. Approval of the institutional ethics committee was obtained prior to this study.

### Study plan

According to OTA/AO classification two pre-fractured specimens were matched and 50:50 randomized by flipping a coin to be operated via an anterolateral approach in supine positioning or a modified posterolateral approach in prone position. Positioning during surgery was secured by attaching the proximal femur shaft to a clamp allowing for 30° rotation. The preparation time before reduction was recorded and thereafter performed using standard reduction techniques and provisional fixation by k-wires and reduction clamps. Reduction was controlled by fluoroscopy (Cios Spin, Siemens, Germany) until a satisfying result was achieved. Flowing this step, a 3D scan (Cios Spin, Siemens, Germany) was recorded that was used for later assessment of the reduction quality.

### Surgical technique

The anterolateral standard approach was performed [14] in supine position. To gain intraarticular access the meniscotibial ligament in the anterolateral quadrant of the tibia plateau was dissected and varus stress was applied to visualize the articular surface. If there was an additional fracture of the medial plateau an independent posteromedial approach was performed in prone position according to Galla et al. [15] prior to the AL meaning that the specimens were repositioned in supine position after the posteromedial approach was completed.

The modified posterolateral approach was performed in prone positioning according to the description by Frosch et al. [11]. After one skin incision directly at the location of the proximal fibula, a posterior and anterolateral window were dissected in all cases. Via the posterior window the posterior meniscotibial ligament and popliteomeniscal fasciculi were dissected to gain access to the posterior boarder of the tibia plateau. If a posteromedial approach was necessary this was performed prior to the PL approach, but with the patient staying in prone positioning meaning that no repositioning was done.

Fixation was achieved by k-wires and lag screws. No final fixation was performed in this study on the assumption that final plate fixation will not change the reduction quality when satisfying reduction was achieved with the beforementioned devices.

### Postoperative analysis

Data of 3D scans were pseudonymized and evaluated by a senior orthopedic trauma resident. Reduction quality was assessed based on multiplanar reconstructed scans following different steps of reduction. Fracture steps of the articular surface as well as fracture gaps were analysed at the posterior border of the PLC/PLL segment, crossing of the antero-latero-central (ALC)/antero-latero-lateral (ALL) and PLC/PLL and within the ALC segment. Medial-to-lateral width of the tibial plateau was measured after each reduction step at its widest diameter.

### Statistical analysis

Data are presented as means and standard deviations (SD). The calculation was based on two groups: (1) anterolateral approach; (2) posterolateral approach. Differences between the groups were calculated with an unpaired *t* test and the Wilcoxon rank test for non-parametric parameters. Analysis was performed using GraphPad Prism 8 (San Diego, CA, US). A *p* value < 0.05 was considered significant.

## Results

### Specimen characteristics (Table 1)

**Table 1:** Matched pair analysis of pre-fractured specimens: 10-segment-classification

Specimens	Approach	AO/OTA	Lateral segments	Intercondylar	Medial segments
1	PL	41B3.1	ALC, ALL, PLC, PLL	AC	None
2	AL	41B3.1	ALC, ALL, PLC	AC, PC	None
3	PL	41B3.1	ALC, PLC	AC, PC	None
4	AL	41B3.3	ALC, ALL, PLC, PC	AC, PC	None
5	PL	41C3.3	ALC, PLC	AC, PC	AMC, AMM
6	AL	41C3.3	ALC, PLL, PLC	PC	PMC, PMM
7	PL	41C3.3	ALC, ALL, PLC	AC, PC	PMC, PMM, AMM, AMC
8	AL	41C3.3	ALC, ALL, PLL, PLC	AC, PC	PMC, PMM, AMM, AMC
9	PL	41B3.1	ALC, ALL, PLC	AC	None
10	AL	41B3.1	ALC, ALL, PLC	AC, PC	None

*PL* posterolateral, *AL* anterolateral, *AO/OTA* classification system; 10-segment classification: *AMM* antero-medio-medial, *AMC* antero-medio-central, *PMM* postero-medio-medial, *PMC* postero-medio-central, *AC* antero-central, *PC* postero-central, *ALL* antero-latero-lateral, *ALC* antero-latero-central, *PLL* postero-latero-lateral, *PLC* postero-latero-central

Five matched pairs according to AO/OTA classification were included. The fracture morphology involved the ALC and PLC in all specimens.

### Preparation time and final fracture reduction

Preparation time was significantly different for both approaches (PL 18.2 ± 2.4 min vs. AL 8.6 ± 1.9 min; *p* = 0.0001). For the PL approach 13.6 ± 5.9 min was needed for the posterolateral window and 11.4 ± 1.9 min for the anterolateral extension. Total time of surgery until the result was considered final was 168 ± 58.9 min using the posterolateral approach versus 128 ± 36.6 min using the anterolateral approach (*p* = 0.23).

### Radiographical reduction accuracy (Table 2)

**Table 2:** Pre-fractured specimens: reduction quality

Area of reduction	PL approach (prone)	AL approach (supine)	*p* value
Relative fragment correction (in mm preoperative fracture step vs. ORIF after fluoroscopy)			
PLC/PLL segment	4.3 ± 2.7	0.9 ± 0.5	0.0248
intersegmental area^a^	3.8 ± 5.5	2.1 ± 2.5	0.5503
ALC segment	4.2 ± 4.2	1.3 ± 1.3	0.1709
Fracture step (in mm) after ORIF with fluoroscopic control and reduction quality assessment using a 3D scan			
PLC/PLL segment	0.4 ± 0.7	2.1 ± 1.4	0.0472
intersegmental area^a^	1.6 ± 0.5	2.4 ± 0.5	0.0246
ALC segment	2.2 ± 1.0	2.0 ± 1.5	0.8606

^a^Crossing of the ALC/ALL and PLC/PLL segment; *PL* posterolateral approach, *AL* anterolateral approach, *ORIF* open reduction and internal fixation;10-segment classification: *ALL* antero-latero-lateral, *ALC* antero-latero-central, *PLL* postero-latero-lateral, *PLC* postero-latero-central

Following ORIF and fluoroscopic-guided control, articular surface irregularity > 2 mm was remaining in the ALC, ALC/PLC intersegmental area and PLC/PLL using the AL approach, which was significantly different from the PL approach that achieved reduction < 2 mm in the PLC/PLL and ALC/PLC intersegmental area, but not the ALC. Using the AL approach fracture reduction quality within the ALC, PLC and PLL segment was inferior compared to the modified PL approach.

## Discussion

The results of this study corroborate the concept that direct visualization and accessibility of the fracture site is mandatory to achieve anatomical reduction of tibial plateau fractures. Fractures as type 41-B3 and 41-C3 according to AO/OTA classification frequently include the postero-latero-lateral and postero-latero-central segments that cannot be visualized by the classic anterolateral approach and thereby direct reduction of displaced posterior rim fractures is impossible [2, 3, 10]. Larger remaining fracture irregularities within the ALC, PLC and PLL were apparent in this cadaveric study when using the AL approach with sole fluoroscopic control and less primary reduction was feasible in this area compared to the PL approach.

The findings of this study are in line with recent literature that emphasizes the concept of direct approaches to the lateral plateau to enable sufficient visualization and direct reduction feasibility [12]. Despite many efforts that have been made to scientifically establish this surgical strategy, a comprehensive radiological and clinical outcome study is still missing to validate the concept. Tibial plateau fractures with involvement with the posterolateral column have been demonstrated to significantly hamper the patients clinical outcome, which emphasises the importance of improved treatment strategies in these type of fracture entities [16].

This study was designed to provide an experimental proof of the concept. Therefore, we aimed for optimal reduction in all cases knowing that a surface irregularity of less than 2 mm articular is clinically irrelevant based on the current literature [3, 8]. Importantly, sufficient visualization and direct accessibility independently contribute to the concept of direct approaches. Krause et al. recently illustrated in a cadaveric study the exposure of the tibial plateau depending on the surgical approaches and its extension demonstrating that by using the classic anterolateral approach only 36.6% of the articular surface can be visualized, and can be increased to 65–80% when an osteotomy of the LCL insertion is performed [10, 13]. An additional posterolateral approach provides 19.0% visualization of the articular surface by the posterior window and direct access to the posterior rim and posterior part of the PLC and PLL [10]. In line with the results of this study, Meulenkamp et al. demonstrated in an analysis of postoperative CT scans that insufficient intraoperative visualization is the main failure reason in complex tibial plateau fractures [8]. Direct visualization is key as sole fluoroscopy cannot provide sufficient accuracy for fracture reduction in complex tibia plateau fractures [8, 9, 17, 18]. Intraoperative 3D imaging provides a comparable quality compared to postoperative CT scans and revealed a 26.5% revision rate in tibial plateau fractures, but it is a retrospective examination which has limited value as a reduction tool [18]. A modified posterolateral approach as described by Frosch et al. combines the visualization opportunities of both, the anterolateral and posterolateral approach by the concept of one approach with two windows [10, 11], and provides direct access and manipulation to the posterior part of the PLC and PLL. Importantly, the advantage of the PL in terms of visualization only accounts for the posterior part of the PLC and PLL since the anterolateral proportion of this approach does not differ from the classic AL [13, 19]. If only the anterior window of the PL is necessary, we strongly recommend performing the classic AL since the PL is closer to the peroneal nerve and needs direct visualization including the potential risk of damaging the nerve. The PL can be performed in prone or lateral positioning, which mainly depends on the involvement of the medial condyle. From a technical point of view prone position of the patient allows for at least 270° simultaneous accessibility of the tibial plateau if an additional posteromedial approach is performed, which avoids the need of intraoperative repositioning of the patient and is advantageous in fractures with deeply depressed fracture fragments that impede sufficient reduction. However, the later aspect was not validated in this study. Nevertheless, the modified posterolateral approach is limited in its visualization of the ALC segment [10, 13], which was confirmed by insufficient fracture reduction following fluoroscopy in our study.

In addition to improved visualization of the PLC and PLL when using the PL, a recent study by Jiang et al. demonstrated the relevance of direct accessibility to the fracture site. The study revealed a considerable rate of non-satisfying reduction although an extended anterolateral approach in Schatzker II and V/VI posterolateral plateau fractures was used [20]. Results of this cadaveric study confirmed that optimal reduction less than 1 mm of the PLC and PLL was impossible via an anterolateral approach, which is mainly due to insufficient accessibility. Using the AL approach, displaced posterolateral rim fractures cannot be buttressed and visualized properly [13, 21]. Menzdorf et al. proposed a classification of posterolateral rim fractures and suggested open reduction and fixation in vertical shear fractures with less than 50% bony support of the posterior horn of the lateral meniscus (type 3b) and depression fractures with dislocation > 2 mm and no bony support of the posterior meniscus horn (type 2c) as well as posterolateral fractures with 90° angulated articular surface facing posteriorly (type 1c). However, this classification system was proposed for posterolateral plateau fractures in patients with an ACL injury. Here, the authors reported good clinical results at 18 months of follow-up for arthroscopic and open reduction [7]. We suggest a similar approach in more complex tibial plateau fractures. Importantly, this kind of fracture entities demand a jail technique or variable angulated locking plates for sufficient internal fixation [22, 23]. Alternatively, rim plating has been proposed via a modified anterolateral approach, but it puts the integrity of the lateral collateral ligament under critical risk and cannot be recommended [24].

**Fig. 1:**
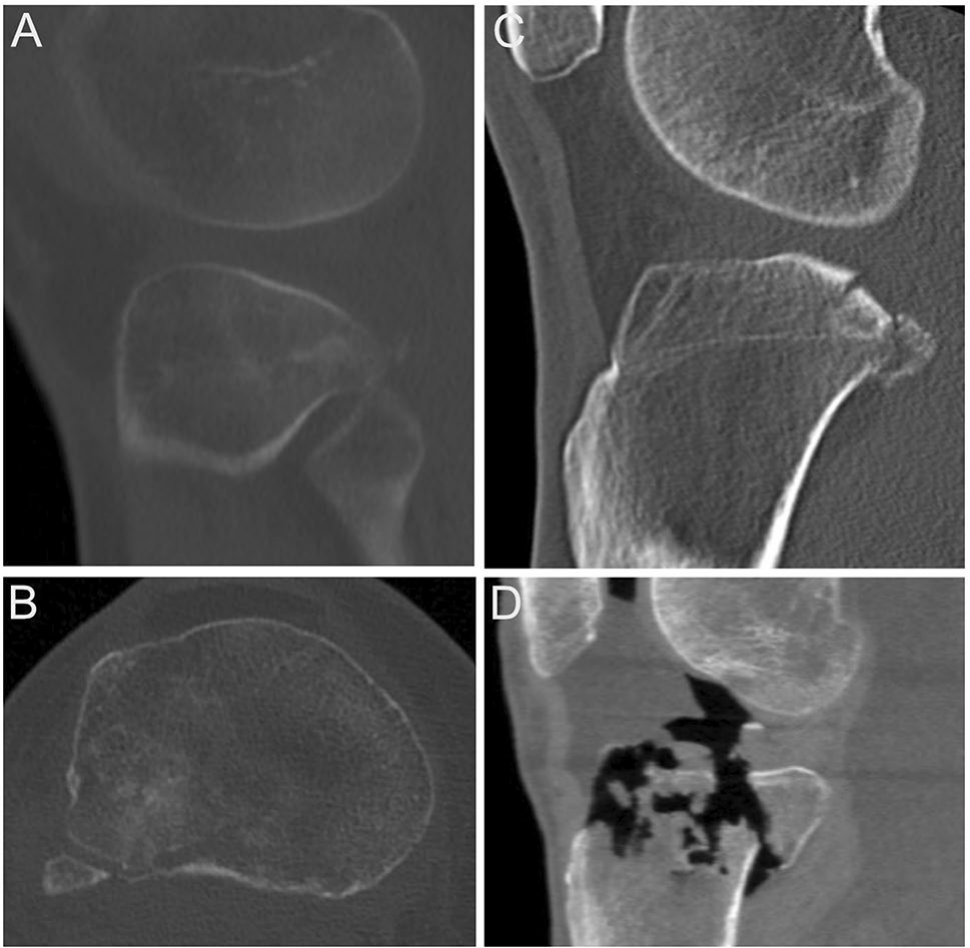
Representative indications for choosing the anterolateral approach (**A**, **B**) or the modified posterolateral approach (**C**, **D**) in tibial plateau fractures involving the posterolateral column. **A**, **B** Impacted and angulated fractures of the PLC/PLL segment can be approached by an anterolateral approach. Depending on the fracture morphology (degree of impaction and angulation) the aimed reduction technique (direct vs. indirect) should be estimated. **C** The modified posterolateral approach enables visualization of the posterior aspect of the PLC/PLL segment, but if the central area of the PLC/ALC segment is involved, additional reductions tools (*arthroscopy or fracturoscopy) or an extended approach becomes necessary. Displaced fractures within the PLC and PLL segment that involve the posterior rim/wall demand direct reduction and visualization that can only be achieved by a modified posterolateral approach. **D** Vertical shear fractures of the PLC/PLL segments can only be visualized and reduced by a posterolateral approach

In summary visualization and need for open accessibility discriminate the indication for choosing the AL or PL (Fig. 1). It may be proposed that if the posterior column is involved, the impaction of the PLC/PLL should be quantified as well as the involvement of the central part of the ALC (PLC/ALC intersegmental area), which results in different indications for using eighter the AL or PL approach:

Modified posterolateral approach:

- deep depression of the PLC/PLL, vertical shear fractures and 90° fracture angulation that necessitates direct reduction and buttressing,
- fracture step within the posterior half of the PLC/PLL close to the intersegmental area,
- bicondylar fractures with the need of simultaneous accessibility of the posteromedial (PM approach) and lateral plateau (PL), which can be realized only in prone positioning of the patient.

Anterolateral approach:

- fracture depression of the ALL, anterior parts of the ALC and lateral parts of the PLL,
- angulated fracture depression of the PLC/PLL that can be indirectly reduced and visualized by additional reduction tools.

Importantly, if the internal part of the PLC/ALC is involved (Fig. 1) both approached fail to adequately visualize the ALC, which necessitated further reduction tools like fracturoscopy or extension of the surgical approaches by an epicondyle or fibula head osteotomy. Fracturoscopy has been demonstrated before to be superior to fluoroscopy and can lead to good clinical results [9, 25].

The scientific conclusions that can be drawn from this study have several limitations and certainly cannot obviate the need for a clinical validation. The fracture morphology of specimens used for AL vs. PL approaches was matched, but it cannot be considered identical, and the number of specimens is too small to overestimate scientific conclusions. In addition, this study focused the lateral compartment and simultaneous treatment of the medial plateau was not scientifically evaluated. To the authors’ experience, complex lateral plateau fractures often present with a coronal medial split fracture that can be addressed via a separate posteromedial approach [26, 27]. However, complex medial plateau fractures will complicate the surgical strategy and need individual concepts, possibly with the necessity to reposition patients during surgery. Due to the absence of bleeding the preparation time and total time of surgery in this study cannot be compared one-to-one to the clinical situation.

## Conclusion

This study provides additional evidence that a direct approach and direct visualization are essential in the surgical treatment of tibial plateau fractures with involvement of the posterolateral-central area. Whether the anterior lateral or the posterior lateral approach is indicated depends on the evaluation of the fracture morphology of the posterolateral column and the intersegmental area.
